# Case Report: Osteosclerotic metaphyseal dysplasia with optic nerve involvement and progressive osteonecrosis of the jaw due to a novel LRRK1 mutation

**DOI:** 10.3389/fendo.2023.1258340

**Published:** 2023-10-18

**Authors:** Chariklia Pieridou, Ataf Sabir, Jonathan Lancashire, Yifan Liang, Kevin McMillan, Nick Shaw, Suma Uday

**Affiliations:** ^1^ Department of Endocrinology and Diabetes, Birmingham Women’s and Children’s Hospital, Birmingham, United Kingdom; ^2^ Department of Clinical Genetics, Birmingham Women’s and Children’s Hospital, Birmingham, United Kingdom; ^3^ Department of Hematology, Birmingham Women’s and Children’s Hospital, Birmingham, United Kingdom; ^4^ Department of Paediatric Palliative Medicine, Birmingham Women’s and Children’s and Community Healthcare Trusts, Birmingham, United Kingdom; ^5^ Department of Oral and Maxillofacial Surgery, Birmingham Women’s and Children’s Hospital, Birmingham, United Kingdom; ^6^ Institute of Metabolism and Systems Research, University of Birmingham, Birmingham, United Kingdom

**Keywords:** skeletal dysplasia, sclerotic dysplasia, osteopetrosis, LRRK1, whole-exome sequencing, osteonecrosis, osteomyelitis

## Abstract

**Background:**

Osteosclerotic metaphyseal dysplasia (OSMD, OMIM 615198) is an extremely rare autosomal recessive osteopetrosis disorder resulting in a distinctive pattern of osteosclerosis of the metaphyseal margins of long tubular bones. To date, only thirteen cases have been reported (eight molecularly confirmed). Five homozygous sequence variants in the leucine-rich repeat kinase 1 (*LRRK1*) gene have been identified to cause OSMD. We present two male siblings with OSMD with a novel *LRRK1* variant.

**Cases:**

The index case, now aged 6 years, was referred aged 9 months when diffuse sclerosis of the ribs and vertebral bodies, suggestive of osteopetrosis, was incidentally identified on a chest radiograph for suspected lower respiratory tract infection. Parents were consanguineous and of Pakistani origin. Further evaluation revealed developmental delay, nystagmus with bilateral optic nerve hypoplasia and severe visual impairment. Skeletal survey confirmed typical changes of OSMD, with widespread diffuse sclerosis and Erlenmeyer flask deformity of long bones. His older sibling, now aged 12 years, was 7 years at the time of referral and had similar clinical course and skeletal findings. Additionally, he had a chronic progressive osteonecrosis of the left mandible that required debridement, debulking and long-term antibiotics. Skeletal survey revealed findings similar to his sibling. Neither sibling had significant skeletal fractures or seizures. Unlike most previous reports suggesting sparing of the skull and lack of visual impairment, our patients had evidence of osteosclerosis of the cranium. Genetic screening for the common autosomal recessive and dominant pathogenic variants of osteopetrosis was negative. Whole Exome Sequencing (WES) followed by Sanger sequencing, identified a novel homozygous *LRRK1* c.2506C>T p. (Gln836Ter) nonsense variant predicted to result in premature truncation of LRRK1 transcript.

**Conclusion:**

Our cases confirm the autosomal recessive inheritance and expand the spectrum of genotype and phenotype of OSMD reported in the literature. Increasing reports of *LRRK1* variants in this phenotype raise the question of whether *LRRK1* should be included in targeted osteopetrosis panels. Bone histology in previous cases has shown this to be an osteoclast rich form of osteopetrosis raising the possibility that haematopoietic stem cell transplantation may be an appropriate treatment modality.

## Introduction

Osteosclerotic metaphyseal dysplasia (OSMD, OMIM 615198) is an extremely rare sclerosing bone dysplasia with a distinctive pattern of osteosclerosis. Since its first description in 1993 (1) only 13 cases have been reported to date (1–9). Iida et al. first reported a homozygous leucine-rich repeat kinase 1 (LRRK1) frameshift deletion in a Moroccan patient with OSMD (5). Downstream of receptor activator of NF-κB (RANK), LRRK1 plays a critical role in regulating cytoskeletal organization, osteoclast activity, and bone resorption and LRRK1-knock out mice display severe osteopetrosis in the metaphysis of the long bones (10). An autosomal recessive pattern of inheritance has been reported. Clinically, the disorder is mainly characterised by skeletal dysplasia and multiple fractures with variable additional phenotypic features including; short stature, developmental delay, hypotonia, seizures, optic atrophy and progressive osteonecrosis of the jaw in the fourth decade of life (1–9). Intra-familial variable expression is noted in reported cases (9). Radiographically, OSMD is characterised by a unique pattern of osteosclerosis, hence making it a distinctive type of osteopetrosis (1). Osteosclerosis is predominantly localised to the metaphyseal margins of the long tubular bones, which show broad sclerotic bands and under-modelling. On the contrary, the diaphyseal bone density is not increased and is often osteopaenic (1, 5, 6). Osseous changes are also evident in the margins of flat bones and to a lesser extent, the metaphyseal equivalents of vertebral end plates, ends of ribs, clavicles and iliac crests. Several reports mention sparing of the skull (1, 5, 6).

Here, we present two siblings of Pakistani descent affected by OSMD secondary to a novel *LRRK1* variant and expand the genotypic and phenotypic spectrum of the disease.

## Case description

### Clinical presentation

#### Patient 1 (P1)

Index case, now aged 6 years, was referred to our team due to dense bones identified incidentally on a chest radiograph at the age of 9 months.

**Figure 1:**
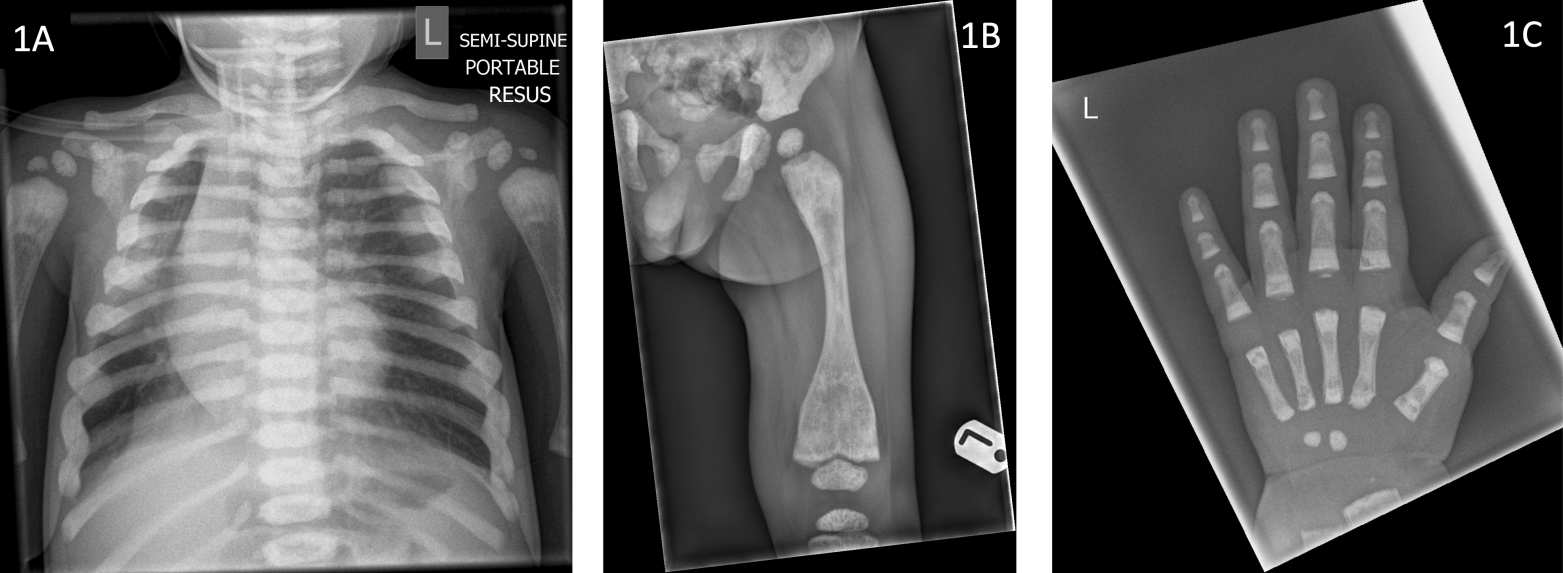
Radiographs of index case (Patient 1) demonstrating **(1A)** sclerotic ribs and vertebrae (aged 9 months), **(1B)** under-modelling of the femur with metaphyseal sclerosis and Erlenmeyer flask deformity (aged 13 months) **(1C)** Metaphyseal sclerosis of the left hand (aged 13 months).

He was born at term by normal vaginal delivery following induction of labour, due to concerns regarding large size and associated maternal hypertension. He presented with respiratory symptoms in the third week of life and required hospitalisation and ventilation support with prolonged antibiotics for a lower respiratory tract infection (LRTI). He had further hospitalisation with recurrent viral LRTIs. At the age of 9 months a chest radiograph, incidentally identified diffuse sclerosis of the ribs and vertebral bodies (**Figure 1**), suggestive of osteopetrosis leading to referrals to paediatric endocrinology and haematology teams.

#### Patient 2 (P2)

Index patient’s brother is a 12-year-old male who was aged 7 years at the time of initial referral following suspected osteopetrosis in the brother. At the time of referral, he was reported to have had nystagmus and strabismus from birth and had developed unilateral blindness. He had experienced recurrent LRTIs as an infant similar to his brother. He had delayed development of motor milestones but was cognitively functioning well.

An MRI scan of the brain identified mild ventriculomegaly, minimal crowding at the foramen magnum with optic nerve hypoplasia and he had abnormal Visual Evoked Potential (VEP). He had developed progressive swelling of the left mandible from age 6 years onwards and a Computed Tomography (CT) scan suggested an initial diagnosis of fibrous dysplasia. He was reviewed by the maxillofacial team and a diagnosis of osteonecrosis of the jaw (ONJ) considered and managed with incision and drainage of the necrotic lesions on 3 occasions. Repeated courses of antibiotics for suspected osteomyelitis were used.

### Family history

Index case was the youngest of three children, born to consanguineous (first cousins) British-Pakistani parents. His older brother aged 7 years had a similar phenotype but a 5-year-old sister was fit and well. There was no history of short stature, developmental delay, fractures, bone or dental problems in either parents or extended family members.

### Further evaluation

**P1:** Further history revealed delayed development. He was unable to sit unaided at 1 year of age and had no words. He had roving eye movements since birth but vision was presumed to be normal. Clinical examination revealed nystagmus, mild hepatosplenomegaly, mild scoliosis and no obvious dysmorphism.

Laboratory examination at the age of 9 months showed full blood count, renal function tests and liver function tests within normal levels. His metabolic bone profile was satisfactory with serum calcium 2.52 mmol/L (2.20-2.70 mmol/L), adjusted calcium 2.45 mmol/L (2.20-2.70 mmol/L), phosphate 1.84 mmol/L (1.30-2.40 mmol/L), alkaline phosphatase (ALP) 323 IU/L (80-330 IU/L), albumin 46 g/L (30-53 g/L), urea 2.3 mmol/L (1.0-5.5 mmol/L) and creatinine 10 μmol/L (14-34 μmol/L). At 28 months he had a haemoglobin level of 110 g/L (110-125 g/L), white blood cells 11.3 x10^9^/L (5-16 g/L) and platelets 351 x10^9^/L (150-400 x10^9^/L).

**Figure 2:**
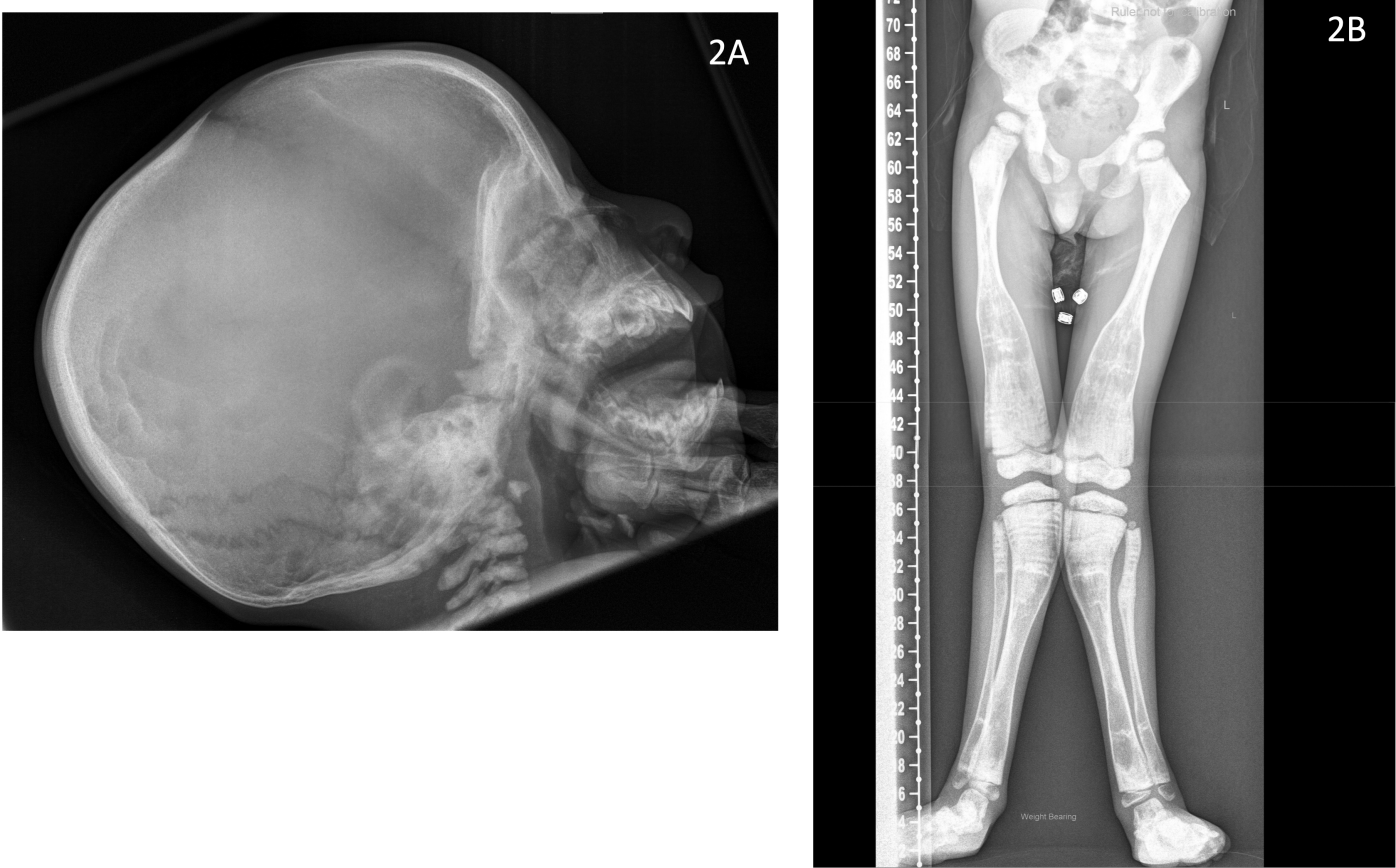
Radiographs of index case (Patient 1) demonstrating **(2A)** diffuse sclerosis of the skull vault, base and the cervical spine (aged 10 months) **(2B)** Valgus deformity with Erlenmeyer flask deformity of the distal femur and proximal tibia bilaterally with metaphyseal sclerosis and evidence of multiple growth arrest lines (aged 5 years, 4 months).

Radiological evaluation revealed sclerotic ribs (**Figure 1A**), Erlenmeyer flask deformity of long bones with sclerotic metaphyses (**Figure 1B**) and dense sclerotic bands were seen in the metaphyses of tubular bones in the hand (**Figure 1C**). Skull radiograph showed diffuse sclerosis (**Figure 2A**). An MRI scan of the brain showed mild prominence of extra-axial cerebro-spinal spaces and lateral ventricles but was thought to be age appropriate. There was bilateral optic nerve/chiasm and tract hypoplasia with no evidence of nerve compression. A visual evoked potential showed normal retinal function but retrobulbar optic pathway dysfunction with severe visual impairment affecting the left eye more than the right. Electro encephalogram (EEG) and auditory evoked potential were normal.

**P2:** Examination revealed, poor growth <0.4^th^ centile, swelling of the left mandible, poor dental hygiene with hypoplastic enamel and valgus deformity of the legs.

Laboratory examination at baseline showed full blood count, renal function tests and liver function tests including ALP within normal levels. His metabolic bone profile was satisfactory with serum calcium 2.39 mmol/L (2.20-2.70 mmol/L), adjusted calcium 2.32 mmol/L (2.20-2.70 mmol/L), phosphate 1.53 mmol/L (1.30-2.40 mmol/L), albumin 46 g/L (30-53 g/L), total vitamin D 75.9 nmol/L (>50 nmol/L), urea 4.1 mmol/L (1.0-5.5 mmol/L) and creatinine 20 μmol/L (14-34 μmol/L).

Skeletal survey displayed similar findings to the brother with involvement of the skull bones. CT scan of the skull revealed uniformly increased density of the skull bones including cervical spine, expansion of internal and external cortices of the ramus and angle of the left mandible, marked mucosal thickening of left maxillary sinus, widening of left osteo-meatal complex and resorption of underlying molar teeth.

### Progress

**P1:** Clinically, he continued to have recurrent LRTIs, requiring hospitalisations, oxygen support and antibiotic treatment until 2 years of age which subsequently improved. He did not sustain any fractures or experience any seizures. Growth continued around -2.3 SDS for height and -2.5 to -2.9 SDS for weight. He was able to stand at 2 years of age and was walking by 3 years but developed progressive skeletal dysplasia with significant valgus deformity (**Figure 2B**), flat feet and ankle inversion by three and a half years of age. Aged 6 years he was able to walk short distances, crawl upstairs and developed early signs of osteonecrosis of the right anterior mandible which is planned to be managed conservatively.

**Figure 3:**
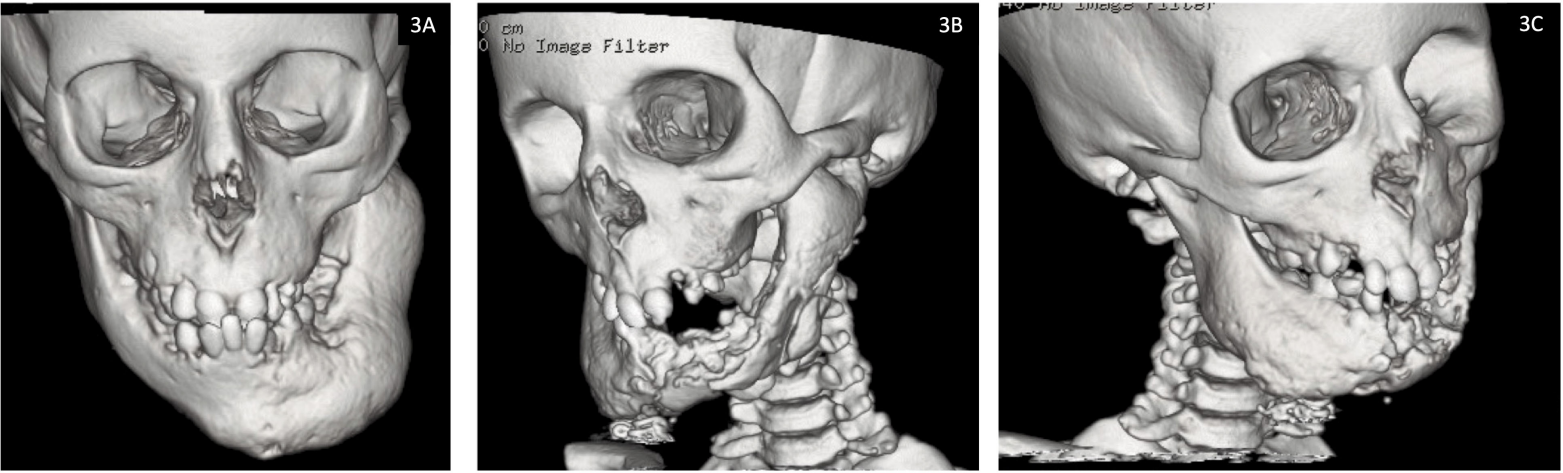
Facial computed tomography (CT) reconstruction images for Patient 2 demonstrating progression of osteonecrosis of the jaw from **(3A)** baseline at 7 years of age and **(3B, 3C)** most recent scan at 12 years of age.

**P2:** Did not sustain any fractures (apart from the mandibular fracture sustained intra-operatively) or experience any seizures. The osteonecrosis has progressed steadily since his first presentation to gradually involve the entire mandible with some mild left mandibular expansion (**Figure 3**). He has had several episodes of osteomyelitis and developed abscesses which were drained and have led to the development of chronic discharging sinuses. The sinuses have increased in size and severity over time. The pathological fracture at the mandibular symphysis was non-healing and necrotic with no viable tissue. There was bone exposure both intra- and extra orally. Most recently further debridement was attempted in the hope of reducing the chronic bleeding from oral mucosa and skin. However, given the severity of the condition, any debridement is likely to lead only to short lived improvement.

More radical treatment including total mandibulectomy has been explored, however, free flap reconstruction is not likely to be successful due to abnormal peripheral skeleton. Free tissue transplant from another donor is not feasible due to concerns about immunosuppression.

Oral intake was limited by the mandibular lesion. Growth remained suboptimal with weight around -3.5 SDS and height around -3.2 SDS. Gastrostomy was inserted to optimise nutrition, aged 12 years.

Mild hepatosplenomegaly was identified with transiently raised alanine transferase and gamma glutamyl transferase aged 12 years, which have normalised. The patient experienced progressive worsening of the osteonecrotic jaw lesion, chronic pain, oozing from the wound, fatigue and nausea. There was no evidence of raised intracranial pressure. He experienced recurrent anaemia requiring frequent (currently monthly) blood transfusion. Other cell lines including granulocytes and platelets remained within normal range. Bone marrow aspirate was considered but felt it would not alter the course. Pain was managed with a combination of paracetamol, morphine, methadone and gabapentin along with tranexamic acid to reduce oozing of blood from the wound.

### Genetic screening

Patient 1’s initial osteopetrosis panel was negative for both autosomal recessive and autosomal dominant pathogenic variants. He was subsequently enrolled in the Genomics England, 100,000 genome project and analysis of the following panels; intellectual disability, skeletal dysplasia and nystagmus, was negative. Subsequent singleton Whole Exome Sequencing (WES) was undertaken via Viapath Genetic laboratories (London) with analysis of skeletal dysplasia panel genes. WES capture was performed using Agilent SureSelectXT Clinical Research Exome (SureSelectXT Human All Exon V5 baited with clinically relevant genes). The enriched exome libraries were sequenced using paired-end, 150 cycle chemistry on an Illumina NextSeq 550. Pathogenicity was assessed using QIAGEN Ingenuity Variant Analysis. WES showed an apparent homozygous *LRRK1* c.2506 C>T p. (Gln836Ter) likely pathogenic variant (NM_024652.6 transcript) which was confirmed by Sanger sequencing and confirmed in his sibling. Pathogenicity was determined according to American College of Medical Genetics (ACMG) criteria (PVS1_strong: nonsense variant predicted to result in premature truncation of the *LRRK1* transcript and PM2; not been reported in gnomAD) (11).

Together these indicated the variant was predicted to be likely pathogenic which was confirmed in conjunction with the phenotype. We were unable to undertake parental studies (to demonstrate parental heterozygosity) or familial analysis in the unaffected sibling of our patients (to establish her genotype), as parents declined genetic screening.

## Discussion

OSMD is a rare entity and the phenotype is still being expanded in the medical literature. Whilst osteosclerosis is the hallmark of the condition, variable phenotypic features have been reported to date, with some cases associated with recurrent respiratory infections, fractures, developmental delay, short stature, bony deformities, osteonecrosis of the jaw, dental abnormalities and visual disturbances. Our reports confirm the autosomal recessive nature of inheritance and the typical skeletal features. We identified a novel LRRK1 gene variant which is the sixth pathogenic variant to be reported in the literature in OSMD. We affirm the involvement of skull and optic nerves which has only been reported in two siblings (9) of the 13 reported cases to date. Progressive and debilitating ONJ, an infrequently (8) reported feature of the condition was a significant finding in one of the two siblings reported here. Contrary to 6 out of the 13 previously reported cases demonstrating one or more fractures (6–9), our patients have not experienced any to date, likely due to limited mobility.

The LRRK1 gene located on chromosome 15q26.3, consists of 34 exons and encodes 2015 amino acids (5, 6). LRRK1 is a multi-domain protein contains leucine-rich and ankyrin repeats in the N-terminal, C-terminal of Roc (COR) and serine-threonine kinase domain, and several C-terminal WD40 domains (5). To date, only five biallelic (homozygous) *LRRK1* variants have been reported in the literature (5–9), accounting for eight affected individuals (**Table 1**) with our patients harbouring the 6^th^ reported variant. The first five cases of OSMD were not molecularly confirmed when published. It is interesting to note that the cases of Moroccan (5), Indian (6) and Palestinian (9) descent all had variants that could lead to residual protein function; where the first two reports (5, 6) had elongated proteins and the latter an inframe deletion. Whereas the Iranian (7) and Bulgarian (8) descent patients and ours all had truncating variants possibly suggestive of a more severe phenotype (as they are more likely to have no residual protein function, except for alternative transcripts). The ONJ in our index case and the ONJ in the Bulgarian patient (8) may point to this more severe phenotype in the truncating variant cases. The ONJ in the Bulgarian adult patient was noted in the third decade of his life and was slowly progressive over a decade (8). However, the ONJ in both our patients started at the age of 6 years and in P2 has progressed with unrelenting aggression since diagnosis. The pattern of disease noted in P2 is very similar but more aggressive than osteonecrosis due to osteopetrosis. Similar aggressive disease is seen with radiation associated osteonecrosis (typically seen in post head and neck radiotherapy) in adults (12). P2 also experienced anaemia which was a feature in the adult patient with ONJ, however there was no report of the adult patient requiring transfusion (8). We speculate that the severe anaemia in our patient requiring transfusion could be multi-factorial related to suboptimal nutrition, oozing of blood from the osteonecrotic wound and potentially encroachment of the sclerotic bone into marrow space, nonetheless the other cell lines remain intact. Granulocytopaenia and hepatosplenomegaly has been previously reported (4).

**Table 1:** Summary table of all molecularly confirmed LRRK1 cases reported in literature.

	Iida et al 2016 (5)	Guo et al 2016 (6)		Miryounesi et al 2019 (7)	Howaldt et al 2020 (8)	Chorin et al 2022 (9)				Current report		
Cases	1	2	3	4	5	6	7		8	9	10	
Age at diagnosis	14 months	14 years	24 years	14 years	34 years	14 years	11.5 years		9 months	9 months	7 years	
Ethnicity	Moroccan	Indian siblings		Iranian	Bulgarian	Arab-Palestinian siblings				Pakistani		
Genetic variant	c.5938_5944delGAGTGGT Frameshift deletion Elongated protein +29AA p.E1980Afs*66	c.5971_5972insG Frameshift insertion Elongated protein p.A1991Gfs*31		c.G2785T, p.E929X Stop-gain variant	c.261G>A Splice-site variant p.Ala34Profs*33	Chr15:101068759 AGGGGCT>A, c.5965_5970del TGGGGC p.Trp1989Gly1990del				c.2506 C>T, p.(Gln836Ter) Nonsense variant		
Zygosity	Homozygous	Homozygous		Homozygous	Homozygous	Homozygous				Homozygous		
Clinical presentation	Failure to thrive, Hypotonia, Psychomotor mental delay, Severe short stature, Hypodontia	Recurrent fractures, Mild facial dysmorphism, Short stature, Acro-osteolysis, Hepatomegaly, Dental crowding	Recurrent fractures, Short stature	Bone pain, Abnormal gait, Fractures, Severe short stature	Progressive ONJ, Osteomyelitis, Recurrent fractures, Short stature, Facial dysmorphism, Kyphoscoliosis, Anaemia, Dental abnormalities	Horizontal nystagmus, Optic nerve atrophy, Recurrent fractures, Osteomyelitis of the jaw, Short stature, Bony deformities, Poor dentition	Fractures, Dental abnormalities	Global developmental delay, Nystagmus, Optic disc atrophy		Recurrent chest infections, Mild developmental delay, Nystagmus, Partially sighted, Mild scoliosis, Mild hepatosplenomegaly		Poor growth, Recurrent chest infections, Nystagmus and strabismus Unilateral blindness, Delayed motor development, Chronic osteomyelitis of the jaw, ONJ Valgus deformity, Poor dentition, hypoplastic enamel
Skeletal Findings Osteosclerosis: *Metaphysis of long> tubular bones* *Metaphysis of short tubular bones* *Epiphyseal margins of long bones* *Margins of flat bones* *Vertebrae endplates* *Skull* Diaphyseal osteopenia Under-modelling Sandwich appearance of vertebral bodies Erlenmeyer flask deformity of femurs	+ + + + + - - + + n.d.	+ + + + + + - + + +	+ + mild + + + - - + mild + n.d.	+ - + - + - - n.d. + +	+ + + + + - n.d. + + +	+ + + + + + - + + n.d.	+ - + - - - - + + n.d.	+ - + - - - - + + -		+ - - - - + (diffuse) - - - +		+ + + - - + (diffuse) - - - +
Investigations *Blood/urine analysis* *Radiographic examinations* *Other*	↓ ALP InvestigationPlain films n/a	Normal Plain films n/a	n/a Plain films n/a	↑ CRP Plain films, MRI, PET n/a	Mild anaemia Plain films Bone biopsy, Osteoclast differentiation	n/a Plain films, CT head Ophthalmological examination	n/a Plain films Ophthalmological examination	n/a Plain films Ophthalmological examination		Normal Skeletal survey, MRI brain; hypoplastic optic nerves/chiasm/tracts Normal EEG/AEP, VEP; retrobulbar optic pathway dysfunction		Anaemia, ↑ RC/CRP, Transient ↑ALT/GGT Skeletal survey, CT head; thickened calvarium VEP, Bone biopsy

AEP, auditory evoked potential; ALP, alkaline phosphatase; ALT, Alanine Transferase; CRP, C-reactive protein; EEG, electroencephalogram; GGT, Gamma-glutamyl transferase; n.d., not determined; n/a, not available; ONJ, osteonecrosis of the jaw; RC, reticulocyte count; VEP, visual evoked potential.

In OSMD, bone biopsy has demonstrated an increased bone mass and slightly elevated osteoid levels in the setting of markedly higher osteoclast surface per bone surface compared to reference value (8). The phenotype and bone histomorphometry in OSMD is similar to an osteoclast rich type of osteopetrosis. Patients with osteopetrosis present with dense sclerotic bones, fractures, neurological symptoms with predominantly optic nerve impairment due to compression, bone marrow failure, infections and early death and poor quality of life requiring frequent blood and platelet transfusions, surgery for dental diseases, nerve and cranial decompression and osteomyelitis in long term survivors (13). Haematopoietic stem cell transplantation (HSCT) is the treatment of choice in patients with malignant infantile osteopetrosis, except in certain patients such as those with neurodegeneration (13). This may be considered in severe forms of OSMD. The potential option of HSCT was discussed with our patients but understandably due to lack of experience in similar cases the parents opted to refrain from it. Until more definitive management strategies are uncovered, management should be supportive using the same principles applied to management of patients with osteopetrosis (14).

## Conclusion

Our cases expand the spectrum of genotype and phenotype of OSMD reported in the literature. Truncating variant may represent a more aggressive form of the disease with osteonecrosis of the jaw. Increasing reports of *LRRK1* variants should promote the consideration of including it in targeted osteopetrosis panels. As it is an osteoclast rich form of osteopetrosis, haematopoietic stem cell transplantation may be an appropriate treatment modality, especially in individuals with debilitating disease including cranial osteosclerosis affecting vision.

## Data availability statement

The original contributions presented in the study are included in the article/supplementary material. Further inquiries can be directed to the corresponding author.

## Ethics statement

Written informed consent was obtained from the individual(s), and minor(s)’ legal guardian/next of kin, for the publication of any potentially identifiable images or data included in this article.

## Author contributions

CP: writing – original draft, writing – review & editing. AS: writing – review & editing. JL: writing – review & editing. YL: writing – review & editing. KM: writing – review & editing. NS: writing – review & editing. SU: data curation, investigation, writing – original draft, writing – review & editing, conceptualization.
